# Supplementary material for: The perception of individuals with low back pain regarding reassuring information: Insights based on physiotherapists messages
Source: PLoS One. 2025 Sep 2;20(9):e0323580. doi: 10.1371/journal.pone.0323580 (PMC12404397; doi:10.1371/journal.pone.0323580)
Supplement: S1 Appendix — (DOCX) [file pone.0323580.s001.docx]

**Imagine that your physiotherapist says the following message to you during a session in relation to your current condition. For each message rate how much, it increases your confidence in the safety of your condition:**

| **#** | **Message number and title** | **Message content** | **Theme** |
| --- | --- | --- | --- |
| 1 | **Common experience** | "Back pain in the lower region is a common occurrence, even among individuals in perfect health. In fact, it's so prevalent that approximately 85% of people will encounter it at some point in their lives." | Reassuring using prevalence and statistics |
| 2 | **Serious causes are rare** | "It is important to note that lower back pain caused by a serious medical condition is very rare. In 99% of cases, treatment is simple without the need for complex interventions." | Reassuring using prevalence and statistics |
| 3 | **No signs of cauda equina** | "According to your examination, you have no symptoms that indicate a serious condition, such as problems with bladder control, numbness in the pelvic area, or foot drop. This means that even if your symptoms are due to a herniated disk, surgery or special treatment is not necessary." | Reassuring using red flags clearance |
| 4 | **Pain resolves with time** | "In most cases, the pain improves on its own within a few days to weeks, and typically no special treatment is needed. I’m confident that your pain will also ease, and we’ll work together to help you feel better soon." | Reassurance based on natural healing of back pain and positive recovery expectations |
| 5 | **Beneficial treatment options** | "To help you recover more comfortably, we have a few options. We can look at modifying your daily routine and any sports you're involved in. I'll also show you some exercises that can be beneficial. And if the pain is bothering you, over-the-counter pain medication can provide some relief. | Reassurance based on treatment strategies |
| 6 | **Common findings ≠ pain** | "Disc bulges or spinal changes are common, even in people who have no pain. Therefore, the presence of these findings in your tests doesn’t necessarily explain the source of your pain." | Reassurance based on interpretation of imaging results |
| 7 | **No concerning signs found** | "Physiotherapists are trained to recognize signs of serious medical conditions. In your examination, no concerning signs were found, so it’s likely that your back pain is not caused by anything dangerous." | Reassuring using red flags clearance |
| 8 | **Pain is multifactorial** | "Our pain system is complex and is influenced by various factors, not just the physical state of the tissues." | Reassurance based on explanation of pain neurophysiology |
| 9 | **Pain intensity ≠ severity** | "The amount of pain you're feeling doesn't always match up with how serious your back problem actually is. Sometimes, people can have really intense pain, but when we do tests, we don't find any major issues. So, even if your pain feels severe, it doesn't automatically mean there's serious damage to your back." | Reassurance based on explanation of pain neurophysiology |
| 10 | **Gradual activity ↓ pain** | "After examining you, I can say that even though you're in pain, I haven't found signs of major damage to your back. My advice is to slowly get back to your usual routine, including work and some moderate exercise. Moving around is key to helping you heal and feel better. I want to stress that getting active again isn't risky - it's actually crucial for your recovery. We'll begin with some gentle activities and then build up based on how you're feeling." | Reassurance based on treatment strategies |
| 11 | **No need for special treatment** | “For most people, lower back pain goes away on its own. Your body has a natural ability to heal, so in many cases, you don't need any special treatment. It just takes some time." | Reassurance based on natural healing of back pain and positive recovery expectations |
| 12 | **Patient autonomy** | "Whether we continue with treatment really depends on you and how much this pain is affecting your daily life. If you feel it would help, we can put together a plan that's tailored to your specific situation and needs." | Reassurance based on treatment strategies |
| 13 | **Imaging findings ≠ pain** | “What we see on X-rays, CT scans, or MRIs doesn't always match up with how much pain you're feeling. Just because something shows up on these images, it doesn't necessarily mean it's causing your pain or that your pain is more severe." | Reassurance based on interpretation of imaging results |
| 14 | **No signs of cancer** | "Based on your examination, there are no signs indicating a serious condition like cancer. You have no history of cancer in the past decade, no unexplained significant weight loss, and your pain changes with body movement.” | Reassuring using red flags clearance |
| 15 | **No signs of disc herniation** | "Back pain is often linked to disc herniation when it's accompanied by severe leg pain, electric shock sensations, or numbness. Since you don't have these symptoms, the likelihood of a disc herniation is significantly reduced." | Reassurance based on natural healing of back pain and positive recovery expectations |
| 16 | **Imaging not needed** | "Lower back pain lasting less than a month typically doesn't require a CT or MRI scan unless specific medical indicators are present. In your case, no such signs were found during the examination, so imaging is not necessary at this time." | Reassurance based on interpretation of imaging results |
| 17 | **Disc herniations natural healing** | "Most disc herniations heal on their own within a few months, and I expect that you will recover and return to your normal activities soon as well." | Reassurance based on natural healing of back pain and positive recovery expectations |
| 18 | **No signs of infection** | "Based on your examination, there are no signs indicating a serious condition like an infection. You don’t have a high fever, chills, or night sweats, which is reassuring." | Reassuring using red flags clearance |
| 19 | **Age-related changes** | "Back imaging often reveals changes that are part of normal aging, much like gray hair or wrinkles. These findings don't necessarily signify a medical issue or cause pain. They're simply the body's natural progression over time." | Reassurance based on interpretation of imaging results |
| 20 | **No signs of fracture** | "Based on your examination, there are no signs suggesting a fracture in your back. You haven’t experienced significant trauma, such as a fall from a height, a direct blow to the back, or a car accident. Additionally, you don’t have any conditions or take medications that would increase the risk of osteoporosis." | Reassuring using red flags clearance |
| 21 | **Safety netting** | "To reassure you, here’s a list of symptoms that would require further medical evaluation:  Fever, chills, or a general feeling of illness accompanying back pain.  Numbness, loss of sensation, or decreased sensation in the lower body, such as the pelvic area or genitals.  Weakness, foot drop, or abnormal sensations in the legs.  Difficulty controlling the bladder or bowel (such as difficulty starting urination, inability to stop urine, or loss of bladder or bowel control).  Significant worsening of the pain, especially at night or during rest.  Major and unexplained weight loss." | Reassuring using red flags clearance |

| **Messages** | **Theme** |
| --- | --- |
| 1 & 2 | Reassuring using prevalence and statistics |
| 3, 7, 14, 18, 20 & 21 | Reassuring using red flags clearance |
| 4, 11, 15 & 17 | Reassurance based on natural healing of back pain and positive recovery expectations |
| 6, 13, 16 & 19 | Reassurance based on interpretation of imaging results |
| 5, 10 & 12 | Reassurance based on treatment strategies |
| 8 & 9 | Reassurance based on explanation of pain neurophysiology |
