# Supplementary material for: The perception of individuals with low back pain regarding reassuring information: Insights based on physiotherapists messages
Source: PLoS One. 2025 Sep 2;20(9):e0323580. doi: 10.1371/journal.pone.0323580 (PMC12404397; doi:10.1371/journal.pone.0323580)
Supplement: S2 Supplementary file — (DOCX) [file pone.0323580.s002.docx]

| Correlation of messages ratings with background variables and patient personality characteristics | | | | |
| --- | --- | --- | --- | --- |
| **Variable** | **Message** | **Correlation (Spearman’s rho)** | | **P-value** |
| Age | *Serious causes are rare* | -0.139 | | 0.001 |
| Age | *No signs of cauda equina* | 0-0.09 | | 0.037 |
| Age | *Gradual activity ↓ pain* | -0.096 | | 0.025 |
| Age | *Patient autonomy* | -0.097 | | 0.025 |
| Age | *No signs of disc herniation* | 0-0.13 | | 0.002 |
| Symptoms duration | *Pain resolves with time* | -0.155 | | 0.000 |
| Symptoms duration | *No need for special treatment* | -0.182 | | 0.000 |
| NPS | *Common experience* | -0.085 | | 0.046 |
| NPS | *No signs of cauda equina* | -0.094 | | 0.029 |
| NPS | *Pain resolves with time* | -0.089 | | 0.037 |
| NTS | *Pain is multifactorial* | -0.085 | | 0.048 |
| NTS | *Gradual activity ↓ pain* | 00-0.1 | | 00.02 |
| NTS | *No need for special treatment* | -0.152 | | 0.000 |
| NTS | *No signs of cancer* | -0.087 | | 0.043 |
| NTS | *No signs of infection* | -0.094 | | 0.029 |
| NTS | *Safety netting* | -0.098 | | 0.022 |
| NPS=Numeric pain scale; NTS=Neuroticism total score | | |  |  |

| Correlation analysis of theme ratings with participant characteristics. | | | | |
| --- | --- | --- | --- | --- |
| **Variable** | **Theme** | **Correlation (Spearman’s rho)** | | **P-value** |
| Age | *Reassuring using prevalence and statistics* | -0.121 | | 0.005 |
| Symptoms duration (weeks) | *Reassuring using red flags clearance* | -0.148 | | < 0.001 |
| Symptoms duration (weeks) | *Reassurance based on natural healing of back pain and positive recovery expectations* | -0.214 | | < 0.001 |
| Symptoms duration (weeks) | *Reassuring using red flags clearance* | -0.148 | | < 0.001 |
| NPS | *Reassurance based on natural healing of back pain and positive recovery expectations* | -0.114 | | 0.008 |
| NPS | *Reassuring using prevalence and statistics* | -0.11 | | 0.010 |
| NTS | *Reassuring using red flags clearance* | -0.113 | | 0.008 |
| NTS | *Reassurance based on treatment strategies* | -0.104 | | 0.015 |
| NPS=Numeric pain scale; NTS=Neuroticism total score | | |  |  |

| Effect Sizes (Cohen’s r) of Post-Hoc Pairwise Comparisons for Education and Imaging Across Themes After a Significant Kruskal-Wallis Test. | | | |
| --- | --- | --- | --- |
| **Theme** | **Comparison** | **Z-value** | **Cohen’s r** |
| *Red flags* | High school vs. Master's degree or Higher | 2.806 | 0.120 |
| *Red flags* | High school vs. Bachelor's degree | 2.177 | 0.093 |
| *Red flags* | Bachelor's degree vs. Master's degree or Higher | -0.942 | -0.040 |
| *Natural healing* | High school vs. Master's degree or Higher | 2.850 | 0.122 |
| *Natural healing* | High school vs. Bachelor's degree | 1.383 | 0.059 |
| *Natural healing* | Bachelor's degree vs. Master's degree or Higher | -1.886 | -0.081 |
| *Pain physiology* | High school vs. Bachelor's degree | 2.435 | 0.104 |
| *Pain physiology* | High school vs. Master's degree or Higher | 1.499 | 0.064 |
| *Pain physiology* | Bachelor's degree vs. Master's degree or Higher | 0.926 | 0.040 |
| *Prevalence and statistics* | CT vs. No imaging | 3.516 | 0.151 |
| *Prevalence and statistics* | MRI vs. No imaging | 2.791 | 0.120 |
| *Prevalence and statistics* | Bone scan vs. No imaging | 2.097 | 0.090 |
| *Natural healing* | MRI vs. No imaging | 4.905 | 0.210 |
| *Natural healing* | CT vs. No imaging | 1.796 | 0.077 |
| *Natural healing* | MRI vs. CT | 2.506 | 0.107 |
| *Natural healing* | X-ray vs. CT | 2.488 | 0.107 |
| *Pain physiology* | X-ray vs. CT | -3.012 | -0.129 |
| *Pain physiology* | MRI vs. CT | 2.944 | 0.126 |
| *Pain physiology* | X-ray vs. No imaging | 2.176 | 0.093 |

All effect sizes are significant at p < 0.005.

*Prevalence and statistics* = Reassuring using prevalence and statistics; *Red flag =* Reassuring using red flags clearance; *Natural healing =* Reassurance based on natural healing of back pain and positive recovery expectations; *Imaging =* Reassurance based on interpretation of imaging results; *Treatment strategies =* Reassurance based on treatment strategies; *Pain physiology =* Reassurance based on explanation of pain neurophysiology
